# An SiO_2_-Filled Matrix to Improve the Thermal Behavior and Surface Integrity of Fiber-Reinforced Polymers Under Dry Milling

**DOI:** 10.3390/polym18060698

**Published:** 2026-03-13

**Authors:** Ali Mkaddem, Makram Elfarhani, Brahim Salem, Yousef Dobah, Yousof Ghazzawi, Abdessalem Jarraya

**Affiliations:** 1 Department of Mechanical and Materials Engineering, Faculty of Engineering, University of Jeddah, Jeddah 21589, Saudi Arabia; ydobah@uj.edu.sa (Y.D.); ajarraya@uj.edu.sa (A.J.); 2 LA2MP, National School of Engineering of Sfax, University of Sfax, Sfax 3038, Tunisia; makram.farhani@gmail.com (M.E.); salemiset@gmail.com (B.S.); 3 SABIC Plastic Applications Development Center, SABIC, Riyadh 11514, Saudi Arabia; ghazzawiy@sabic.com

**Keywords:** glass fiber, SiO_2_, milling, temperature, heat, SEM, surface integrity

## Abstract

This study discusses the thermal behavior of glass fiber-reinforced SiO_2_-filled polymers in dry milling. Focus is put on the effects of the addition of SiO_2_ particles on cutting-generated heat and the fresh-surface integrity of the composite. Cutting trials were developed using a Spinner U-620 5-axis CNC machine. Real-time temperature histories owing to the dry milling of both Glass/Epoxy and Glass/Polyester composites were recorded using thermocouples preinstalled within the composite specimen. SEM inspections were conducted to elucidate the prevailing failure mechanisms during the material removal process. The results showed that fiber orientation significantly dominated thermal responses. Cutting perpendicular to the fiber orientation results in a critical temperature, while the addition of SiO_2_ particles effectively reduces the temperature overlaps and peak values, causing the temperature to drop. The addition of SiO_2_ serves to keep the temperature sufficiently lower than the glass transition point of the matrix. However, increasing the feed rate from 50 mm/min to 150 mm/min reduced the overall temperature during cutting. Under similar cutting conditions, Glass/Polyester composites exhibited lower peak temperatures and heat quantities than Glass/Epoxy regardless of the feed rate and fiber orientation. Observations revealed that fiber orientation and matrix type strongly influence the intensity of the thermal and mechanical damages induced. These findings suggest that the addition of silicon dioxide can adjust the thermal balance in dry cutting and may improve the composite’s structural integrity significantly. Such a composite design promotes the heat control of sensitive parts in advanced engineering applications.

## 1. Introduction

Polymer resin is commonly used as a matrix material in glass fiber-reinforced polymers (GFRPs). The combination of glass fibers and polymer resin results in a composite with excellent mechanical properties, such as high specific tensile strength, good impact resistance, and low weight [1]. Due to these advantages, GFRP composites are widely used across various engineering sectors, such as the aerospace, automotive, and construction industries [2]. Machining operations on GFRP are typically required to achieve functional surfaces that cannot be easily produced through the shaping process [3]. GFRP has low thermal conductivity, mainly due to the properties of both the resin and fiber. Consequently, the heat produced during machining tends to remain concentrated in the cutting zone, rather than dissipating throughout the workpiece. This localized heat becomes an issue if the cutting temperature exceeds the resin’s glass transition temperature (T_g_), causing matrix degradation and potentially altering the material’s mechanical performance [4,5]. Fillers such as silica, graphite, fly ash, titanium dioxide, and alumina [6] and nanofillers such as graphene, CNTs, and interleaves [7,8,9] have become increasingly popular because of their ability to enhance the thermomechanical properties of GFRP composites, which act to improve their behavior during cutting. The estimation of heat energy becomes crucial to achieve a high-quality surface finish since excessive heat causes thermal degradation of the matrix, leading to reduced structural integrity and reduced precision. Thus, effective heat management through the optimization of cutting parameters ensures the preservation of material properties and enhances the overall performance of the milling process.

The milling of composites remains a subject of ongoing uncertainty for both industry professionals and researchers. Several researchers have examined how process parameters affect the machining of composite materials. The milling machinability of GFRP has been the focus of various studies [10]. The most important cutting parameters in milling fiber-reinforced polymer (FRP) are cutting speed, depth of cut, and feed rate [11]. Hocheng et al. [12] investigated the impact of feed rate and fiber orientation on cutting quality, cutting forces, and chip formation. They categorized the chips into three types: powder; ribbons, which arise from fiber fracture; and buckling. Kumar et al. [13] examined the surface finish, thrust forces, and machinability index when milling GFRPs with a four-fluted HSS end mill. Their findings revealed that cutting speed had a major influence on the surface finish, especially in 0°/90° fiber orientations. Su et al. [14] explored how the cutting depth affects chip formation and subsurface damage during the orthogonal cutting of carbon fiber-reinforced polymers (CFRPs). The results revealed that the cutting depth plays a crucial role in chip formation, particularly for fiber orientations of 90° and 120°. Furthermore, as the cutting depth increases, the depth of subsurface damage also increases. The material removal mechanism changes considerably with different fiber orientations in the GFRP milling process. This variation in fiber orientation likely accounts for the differing cutting forces experienced during machining, which can result in lower laminate layer production quality and cause manufacturing defects [13]. Geier et al. [15] investigated the cutting forces during the milling of CFRP material with an end mill, focusing on different fiber orientations and cutting directions. The results showed that the fiber orientation in the CFRP structure plays a crucial role in influencing the cutting forces. Voss et al. [16] experimentally explored how fiber orientation, tool geometry, and machining parameters influence surface quality and tool life. Nguyen et al. [17] reported that CFRP plies oriented at 45° produced significant flank wear because the fibers undergo bending before being cut, which results in springback, forcing the fibers to come into contact with the flank face, generating significant wear in that tool region. However, when cutting perpendicular to the fiber orientation, neat mode-II fiber failure dominates the material removal process. Rough fiber sections act directly on the tool-tip, inducing pronounced tool edge rounding. In contrast, plies with a 0° fiber orientation exhibit the least tool wear since fiber/matrix debonding dominates the material removal process.

Cutting heat is an inevitable phenomenon that occurs during the machining process and is a crucial factor that directly impacts the quality of the machined parts. There is limited research on the temperature during GFRP milling. If the milling temperature exceeds the glass transition temperature of the thermosetting matrix resin, the resin on the machined surface or surface layer may degrade. Yashiro et al. [18] studied the cutting temperatures in the milling process by utilizing a tool–workpiece thermocouple and an embedded K-type thermocouple in the workpiece. Their results indicated that, at higher cutting speeds, the temperature at the cutting point has a minimal and restricted influence on the machined layer. Nor Khairusshima et al. [19] extended tool life by lowering the temperature in the cutting region using cold air at −10 °C. Wang et al. [20] examined how process factors impact cutting temperature when milling CFRP composites. They reported that cutting speed was the most significant parameter influencing milling temperature, followed by feed rate and cutting depth. More recently, Salem et al. [21] studied the sensitivity of tool-tip temperature to cutting speed in GFRP/Al/GFRP and Al/GFRP/Al arrangements. It was found that the peak temperature recorded when engaging Al/GFRP/Al interfaces reaches 119.7 °C under critical cutting speeds, which is over the glass transition point of the matrix (T_g_ ~110 °C). However, when drilling GFRP/Al/GFRP, the tool temperature does not exceed 107 °C. 

Bondioli et al. [22] investigated epoxy–silica nanocomposites, focusing on their preparation, experimental characterization, and modeling to determine the relationship between microstructure and mechanical performance. Complementarily, Mohammad et al. [23,24] advanced the field of thermally conductive polymer nanocomposites by developing scalable fabrication methods for 3D BN-based PVA aerogels with nacre-like layered architectures and reviewing recent progress in thermally conductive 3D aerogels and foams with segregated nanofiller frameworks. These studies contribute valuable insights into the design of multifunctional nanocomposites with enhanced thermal and mechanical properties for advanced engineering applications. Ilangovan et al. [25] discovered that incorporating silica nanoparticles (SiO_2_) enhances the thermal properties of FRP composites. Zeng et al. [26] reported a 20–30% enhancement in the fracture toughness (GIC) of carbon fiber/epoxy composites by adding 6 wt% SiO_2_ nanoparticles. While the sensitivity of mechanical performance to the addition of fillers has been proven [7,8,9,26], their effects on heat balance through the composite structure still remain understudied.

This study quantifies the thermomechanical severity of dry milling in GFRP by combining in situ temperature measurements close to the trimmed surface with an energy-based thermal evaluation of cutting-generated heat. Unlike prior works that typically isolate either machining parameters or material modifications, the present investigation integrates (i) matrix type (epoxy vs. polyester), (ii) fiber orientation (0°/90°), (iii) feed rate, and (iv) SiO_2_-filled matrices, and further correlates the thermal response with observed surface integrity (SEM). The resulting dataset highlights how SiO_2_ fillers can be used as a material-level lever to reduce thermal loading and mitigate surface damage under severe dry milling conditions.

## 2. Materials and Methods

### 2.1. Materials

To investigate the effects of silica particles, matrix type, fiber orientation, and feed rate on the temperature and heat energy generated during the milling process, various specimens were prepared. In a first series, pure-epoxy and pure-polyester specimens were first prepared. They are used later as references for investigating heat evolution. The second series concerns the fabrication of Glass/Epoxy and Glass/Polyester specimens. The last series, however, concerns the preparation of SiO_2_-filled composite specimens by introducing silica sand particles. Typically, Glass/(Epoxy+SiO_2_) and Glass/(Polyester+SiO_2_) composites are used to assess the influence of silicon dioxide on cutting behavior and, particularly, on temperature generation.

The epoxy resin used in this study was provided by RESOLTECH Co. (Rousset, France) under commercial reference 1050/1055S. It was manually prepared by mixing the hardener 1055S with the resin 1050 in a 1:3 ratio. As for the polyester resin, it was provided by TURKUAZ POLYESTER Co. (Kocaeli, Turkey) with commercial reference TP280. For curing, methyl ethyl ketone peroxide (MEKP) was used as the catalyst. Table 1 summarizes the properties of the polymer matrices considered in the preparation of the different specimens.

Unidirectional E-glass fibers with an areal density of 530 g/m^2^ and an effective density of 2.55 g/cm^3^ were supplied by SICOMIN (Châteauneuf-les-Martigues, France). SiO_2_ silica particles provided by TECNOPOL Co. (Barcelona, Spain) were used to enhance the toughness of both epoxy and polyester resins. Table 2 presents the typical properties of the glass fiber and SiO_2_ particles used in this study. The specimens were cut from the composite panel with 80 mm × 40 mm × 8 mm dimensions.

The test specimens were designed to fit with the experimental setup and to offer enough flexibility for the preparation of the thermocouple (TC) locations. The composite panel thickness and flatness were systematically checked after molding, since both parameters can critically influence the temperature histories during milling.

Because of the significant difference in thermal conductivities, the heat flowing into or from the workpiece depends on whether the material is cut parallel or perpendicular to the fibers. The thermal conductivities of the composite in the fiber direction ($\lambda_{∥}$) and perpendicular to the fiber direction ($\lambda_{⊥}$), can, respectively, be calculated as follows:

(1) $$\lambda_{∥}=V_{f}\lambda_{f}+V_{m}\lambda_{m}+V_{s}\lambda_{s}$$

and

(2) $$\frac{1}{\lambda_{⊥}}=\frac{V_{f}}{\lambda_{f}}+\frac{V_{m}}{\lambda_{m}}+\frac{V_{s}}{\lambda_{s}}$$

or

(3) $$\lambda_{⊥}=\frac{\lambda_{f}{\cdot \lambda }_{m}{\cdot \lambda }_{s}}{V_{f}\left(\lambda_{m}\lambda_{s}\right)+V_{m}\left(\lambda_{f}\lambda_{s}\right)+V_{s}\left(\lambda_{f}\lambda_{m}\right)}$$

where $\lambda_{f}$, $\lambda_{m}$, and $\lambda_{s}$ are the thermal conductivities of fiber, matrix, and silicon dioxide, respectively, and $V_{f}$, $V_{m}$, and $V_{s}$ are the associated volume fractions. Using Equations (1) and (3), the conductivities of the considered composites are calculated according to the data in Table 1 and Table 2. The obtained values are given in Table 3.

### 2.2. Specimen Preparation

To prepare epoxy and polyester panels, a compression molding process was applied. The pure epoxy was first mixed with 30% 1055S commercial hardener and kept in the mold at room temperature for 24 h, followed by curing at 60 °C for 15 h. The whole cycle was conducted at a uniform pressure of 3 kPa. However, the polyester was mixed with 2% MEKP commercial hardener supplied by RESOLTECH Co. (Rousset, France). Then, it was kept in the mold at room temperature for 2 h and cured at 60 °C for 2 h at the same pressure. After curing, resins were kept inside the oven until room temperature was reached.

To prepare Glass/Epoxy and Glass/Polyester composite panels, a compression molding process was employed. The molding step consists of inserting ten plies of unidirectional fibers embedded within the matrix mixture into the mold. The mold was then closed and subjected to a curing cycle similar to that previously described for the preparation of the resin panels. Subsequently, the composite plates were cut, ensuring that the fibers were oriented at 0° and 90° with respect to the direction of tool advance. The glass fiber volume fraction was fixed to 15% in all specimens.

For the preparation of the SiO_2_-filled composites, silica particles were first mixed with the resin at room temperature and stirred using a mechanical system for approximately 20 min. Then, the hardener was added to the resin–SiO_2_ mixture and stirred for about 5 min. A similar procedure was followed for molding and curing the SiO_2_-free composite. The silicon dioxide fraction (wt%) was fixed to 28.8% in all specimens, while the fiber fraction was kept constant.

### 2.3. Temperature Acquisition System

**Figure 1:**
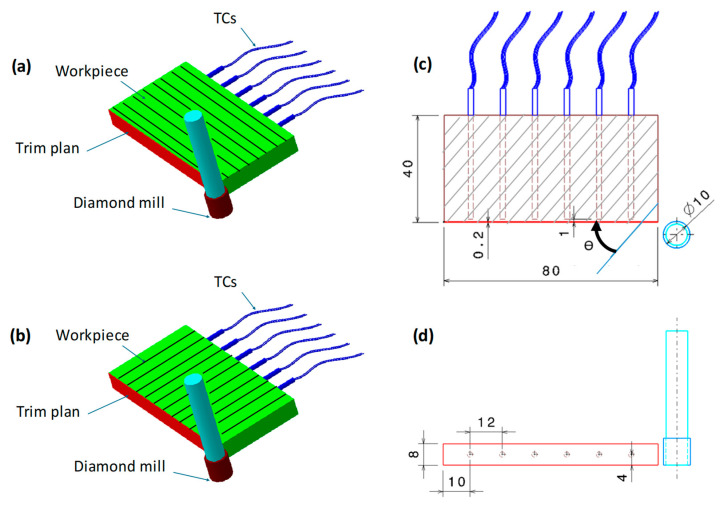
Orthogonal cutting test. (**a**) Milling test parallel to fiber, (**b**) milling test perpendicular to fiber, (**c**) the position of TC sensing heads relative to the trim plan, and (**d**) TC locations along the specimen. All dimensions are in mm.

A temperature measurement strategy was designed to track the real-time evolution of temperatures along the machined surface. As illustrated in Figure 1, Type-K thermocouples were inserted into 2 mm diameter holes on the rear side of the cut surface. The TCs were preinstalled, 12 mm equidistant, within the mid-thickness of the trim plan to determine the temperature evolution 1 mm away from the cut surface (Figure 1), which is low enough to reflect the effective temperature generated at the fresh surface.

During tests, the output voltage signal from each TC was recorded using an Arduino board supplied by ELECTRONIC SHOP Co. (Sfax, Tunisia). The PLX-DAQ version 1 software, communicating with an Arduino application supplied together with the board, is able to process the 200 outputs and handle data transfer. The temperature history recording is ensured at a maximum frequency of 4 Hz. The Type-K thermocouples, provided by ELECTRONIC SHOP Co. (Sfax, Tunisia), were selected due to their high measurement reliability, offering an accuracy of ±1.5 °C and a resolution of 0.25 °C [40]. Accordingly, the standard deviations of three repeated tests are indicated in the captions of each figure showing the temperature measurements.

### 2.4. Physical Model and Heat-Generation Framework

The milling configuration used in this work can be idealized as a micro-grinding-like process in which multiple diamond abrasive grains intermittently engage the composite. The mechanical power input is dissipated through (i) frictional sliding/rubbing at the tool–work contact, (ii) matrix deformation and shearing, and (iii) fiber fracture and fiber–matrix debonding. Under dry conditions, most of this energy is converted into heat concentrated near the cutting zone. The generated heat is then partitioned between the workpiece, the tool, and the removed chips, while heat conduction inside the composite is governed by anisotropic thermal properties (along-fiber and transverse). The embedded thermocouples positioned close to the trimmed surface provide a transient thermal response that is representative of the near-surface region affected by milling. The cutting-generated heat stored in the workpiece is evaluated from the measured temperature histories using an energy-balance approach, with robust differentiation obtained through polynomial fitting.

### 2.5. Milling Tests Spinner U-620 5-axis

**Figure 2:**
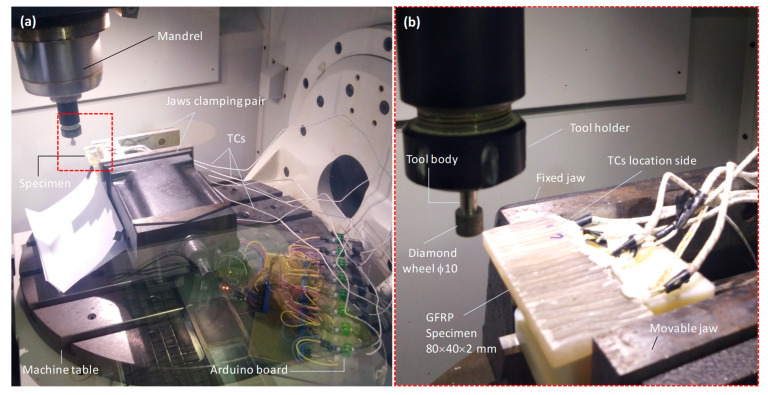
Experimental setup. (**a**) Milling machine with acquisition system, (**b**) enlarged view showing the diamond wheel, the specimen, and the TC position.

Milling tests were performed using a High Speed CNC Machine from ISET Institute (Sidi Bouzid, Tunisia) with a maximum spindle speed of 12,000 rpm, capable of delivering up to 19 kW of power. Figure 2 presents the experimental setup, detailing the tool mounting, test specimen arrangement, and the temperature acquisition system.

The milling operation was conducted with a custom diamond grinding tool, galvanic nickel-bonded, of 10 mm in head diameter, 50 mm in length, and 10 mm in effective cutting height. Milling trials were conducted both parallel (θ = 0°) and perpendicular (θ = 90°) to the fiber orientation, as thermal conduction was expected to be influenced by the fiber orientation. The cutting tests use two different feed rates, namely, 50 and 150 mm/min, a constant cutting speed of 283 m/min and a constant depth of cut of 0.2 mm. All cutting trials are performed under dry conditions. The temperature evolutions arise from a series of six successive recordings.

### 2.6. SEM Analyzes

SEM inspections were performed to examine the surface integrity of the machined surface. Typically, four reference samples were investigated: as-molded matrix, as-machined matrix, as-machined SiO_2_-free composite, and as-machined SiO_2_-filled composite. The inspections were performed on meticulously prepared samples using a TS Quanta 250 microscope from ETAP Center (Tunis, Tunisia). Images were acquired at a maximum magnification of 600 and a resolution of 2048 × 1536 pixels. An accelerating voltage of 10–25 kV and a constant chamber pressure of 70 Pa in low-vacuum mode were applied to ensure optimal contrast and the stable imaging of the non-conductive composite surfaces.

### 2.7. Estimation of Heat Energy

Based on the principle of conservation, the energy flowing into the workpiece via the friction effect at the tool–material interface equals the energy flowing from the workpiece, with some negligible loss. The energy dissipated from the workpiece depends, with great sensitivity, on the temperature. To ensure reliable measurements with negligible losses, the TCs are intentionally placed 1 mm behind the fresh surface, i.e., the tool-tip, to record temperature histories in situ. The heat dissipated from the workpiece during the milling process is as follows [39]:

(4) $$\frac{dQ}{dt}=m_{c}c_{p}\left(\frac{dT}{dt}\right)$$

where Q is the cutting-generated heat energy (J), $m_{c}$ is the mass of the composite workpiece (kg), $c_{p}$ is the specific heat ($J\cdot {kg}^{-1}\cdot C^{-1}$), T is the temperature (°C), and *t* is the time (s).

The mass of the workpiece can be obtained from the density of the composite as follows:

(5) $$m_{c}=\rho_{c}v_{c}$$

where $\rho_{c}$ is the composite density, and $v_{c}$ is the volume of the composite workpiece. Since it depends on the fraction of each constituent, the composite density can be obtained as follows:

(6) $$\rho_{c}=\rho_{f}\cdot V_{f}+\rho_{m}\cdot V_{m}+\rho_{s}\cdot V_{s}$$

where $\rho_{f},\;{\;\rho }_{m},\;and\;{\;\rho }_{s}$ are the densities of the fiber, matrix, and silica, respectively, and $V_{f},\;{\;V}_{m},\;and\;V_{s}$ are their respective volume fractions.

Provided the constituent volume fractions are known, the specific heat of the workpiece can also be calculated as follows:

(7) $$c_{p}=c_{pf}\cdot V_{f}+c_{pm}\cdot V_{m}+c_{ps}\cdot V_{s}$$

where $c_{pf},\;{\;c}_{pm}\;\;and\;c_{ps}$ are the specific heats of the fiber, matrix, and silica, respectively.

**Figure 3:**
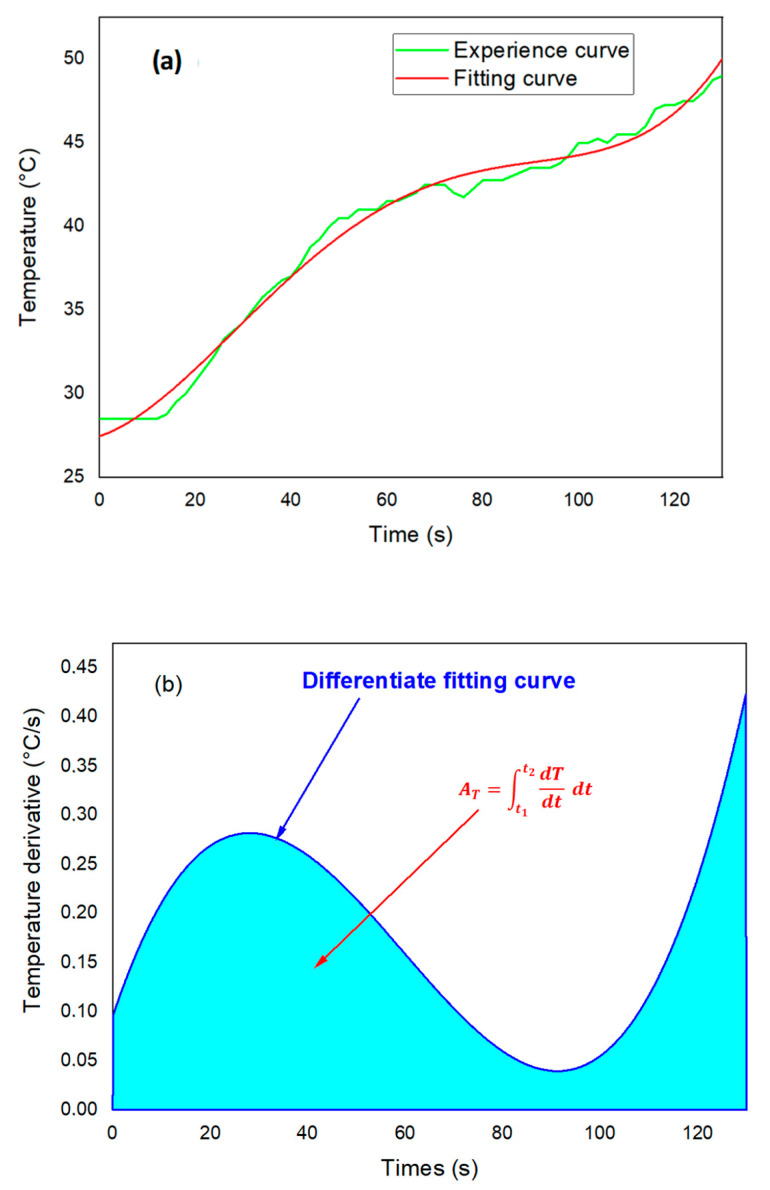
(**a**) T vs.t plot with typical polynomial fitting curves, and (**b**) temperature derivative plot $\dot{T}\;vs.\;t$ obtained from temperature evolution.

To estimate the heat energy generated from the temperature-versus-time curve, the experimental data was fitted by a derivative function. Figure 3a shows an example of experimental data with a fourth-degree polynomial fitting law. Differentiating this polynomial yields the temperature rate $\left(\frac{dT}{dt}\right)$, resulting in a temperature change over time [39].

(8) $$Q=m_{c}\;c_{p}\int_{t_{1}}^{t_{2}}\left(\frac{dT}{dt}\right)dt$$

where $t_{1}$ and $t_{2}$ are the initial and final times of the milling period, respectively. The quantity, $A_{T}=\int_{t_{1}}^{t_{2}}\left(\frac{dT}{dt}\right)dt$, is then calculated as the area delimited by the overlap $\left(\frac{dT}{dt}\right)$ versus time, as shown in Figure 3b.

Table 4 summarizes the mathematical parameters owing to the polynomial fitting of temperature data. Standard deviation (SD), root-mean square error (RMSE), mean absolute error (MAE), and correlation coefficient (R^2^) are specially reported for each cutting test in the design of experiments.

## 3. Results and Discussion

### 3.1. Temperature Overlap When Milling Glass/Epoxy

**Figure 4:**
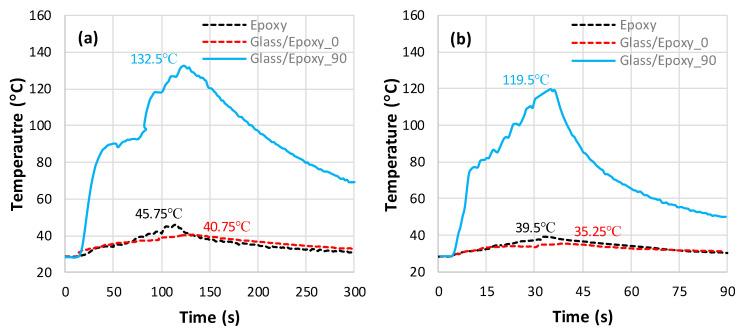
Temperature vs. time obtained when milling fiber-free and fiber-reinforced epoxy. (**a**) f=50 mm/min (0.07 °C ≤ SD ≤ 2.83 °C) and (**b**) f=150 mm/min (1.04 °C ≤ SD ≤ 2.61 °C).

Figure 4 illustrates the temperature-versus-time plots recorded by thermocouples during the milling of a pure-epoxy matrix and a Glass/Epoxy. For composite specimens, the temperature history was recorded when milling the composite parallel and perpendicular to the fiber orientation. Figure 4a shows that when milling perpendicular to the fiber orientation, the temperature increases sharply to reach a peak of 132.5 °C before gradually decreasing in accord with the much lower cooling rate. Meanwhile, the milling of the pure-epoxy matrix and Glass/Epoxy composite parallel to the fiber orientation leads to much lower temperatures, with relatively small differences in peak values, i.e., 45.75 °C and 40.75 °C, respectively.

The results show that milling parallel to the fiber induces less heat than that generated when milling perpendicular to the fiber. This indicates that fiber orientation can significantly impact the workpiece temperature during the machining process. At f=50 mm/min, the peak temperature obtained from milling Glass/Epoxy perpendicular to the fiber (132.5 °C) greatly exceeds the glass transition temperature of the matrix, which approximates 77 °C [41]. This excessive temperature alters the mechanical properties of the epoxy matrix, leading to thermal damage such as matrix burnout and fiber/matrix interface failure. When the feed increases from 50 to 150 mm/min, the peak temperature falls regardless of the material type and fiber orientation, as can be seen in Figure 4b. This decrease is due to the drop in contact time between the workpiece and the tool as the feed rate increases, which results in a decrease in the thermal energy generated by friction [27].

As can be seen in the plots obtained, for both feed rates, the maximum temperature during the milling of the pure-epoxy matrix is slightly higher than that observed during milling of the Glass/Epoxy composite with fibers oriented at 0°. This difference is attributed to the fact that the average thermal conductivity of the pure-epoxy matrix ($\lambda \approx 0.21\;W\cdot m^{-1}\cdot K^{-1}$) is slightly higher than that of the Glass/Epoxy composite in the fiber direction ($\lambda_{∥}\approx 0.18\;W\cdot m^{-1}\cdot K^{-1}$), as highlighted in Table 3. The discrepancies between peak temperatures recorded when milling pure epoxy and Glass/Epoxy parallel to the fiber are too close, i.e., 10.9% for f=50 mm/min and 10.7% for f=150 mm/min. This proves that even feed rate increases influence the temperature histories. However, it does tend to maintain the same increment for milling pure epoxy and Glass/Epoxy parallel to the fiber.

### 3.2. Temperature Overlap When Milling Glass/Polyester

**Figure 5:**
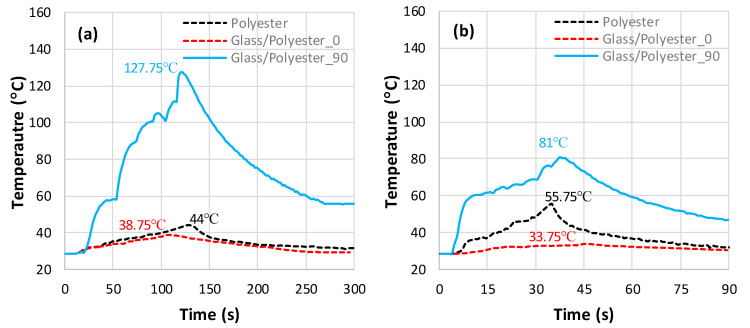
Temperature vs. time obtained when milling fiber-free and fiber-reinforced polyester. (**a**) f=50 mm/min, (0.09 °C ≤ SD ≤ 2.89 °C) and (**b**) f=150 mm/min, (1.14 °C ≤ SD ≤ 2.59 °C).

Figure 5 illustrates the temperature evolution over time obtained during the milling of a pure-polyester matrix and Glass/Polyester composites with fibers oriented at 0° and 90°. The cutting speed and depth of cut were kept constant, as they were for the Glass/Epoxy cutting tests, while two feed rates of 50 mm/min and 150 mm/min were considered.

In Figure 5a,b, we can see a more pronounced effect of the feed rate on the peak temperature compared with the values obtained for the Glass/Epoxy. During the milling of the Glass/Polyester composites perpendicular to the fiber orientation, the peak temperature falls by 46.75 °C when the feed passes from 50 to 150 mm/min. However, the maximum temperatures recorded during the milling of the Glass/Polyester composites are lower than those measured when milling the Glass/Epoxy composites, irrespective of the fiber orientation and feed rate. Only the peak temperature obtained at 150 mm/min for pure polyester, i.e., 55.75 °C (Figure 5b), appears to have increased compared to that obtained for pure epoxy, i.e., 39.5 °C (Figure 4b). Consequently, the discrepancy between the peak temperatures obtained for the Glass/Polyester cut parallel to the fiber and that for pure polyester increases with the feed, in contrast to the discrepancy observed between the peak values for Glass/Epoxy and pure epoxy. Polyester with thermal conductivity approximating that of epoxy does not allow for heat to flow immediately away from the specimen, leading to heat accumulation on the milled surface.

### 3.3. Temperature Overlap When Milling SiO_2_-Filled GFRP

#### 3.3.1. Glass/(Epoxy+SiO_2_)

**Figure 6:**
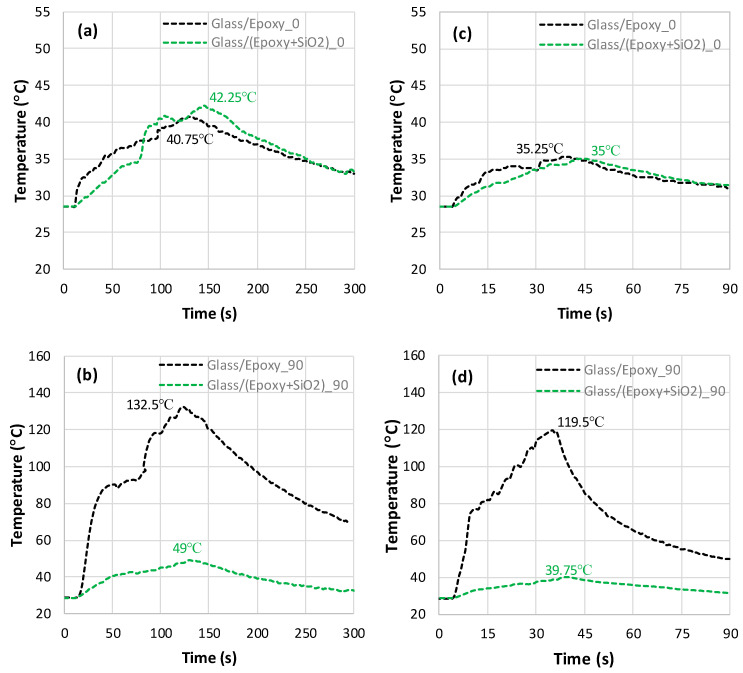
Temperature histories obtained in milling of SiO_2_-free and SiO_2_-filled Glass/Epoxy composites. (**a**) Glass/Epoxy, f=50 mm/min; θ=0°; (0.07 °C ≤ SD ≤ 2.52 °C) (**b**) Glass/Epoxy, f=50 mm/min; θ=90°; (1.57 °C ≤ SD ≤ 2.83 °C) (**c**) Glass/Epoxy, f=150 mm/min; θ=0°; (1.1 °C ≤ SD ≤ 2.04 °C), and (**d**) Glass/Epoxy, f=150 mm/min; θ=90°; (1.36 °C ≤ SD ≤ 2.61 °C).

When milling Glass/(Epoxy+SiO_2_) composites parallel to the fibers (Figure 6a,c), the peak temperature looks relatively low when compared to the peak value recorded when cutting composite perpendicular to the fibers.

According to the findings, the addition of SiO_2_ leads to insignificant peak temperature gaps of about 3.68% and 0.55% for 50 mm/min and 150 mm/min, respectively. Thus, the addition of silica sand serves to limit thermal effects during the milling process regardless of the feed rate. However, the temperature histories exhibit a significant drop when cutting Glass/(Epoxy+SiO_2_) composites perpendicular to the fibers (Figure 6b,d). Typically, the peak temperature decreases by approximately 63% at a 50 mm/min feed rate and 67% at a 150 mm/min feed rate. Overall, incorporating SiO_2_ into the Glass/Epoxy composite is found to be more effective in reducing the maximum temperature when cutting perpendicular to the fibers. This suggests that SiO_2_ plays a crucial role in minimizing heat generation within the material when cutting Glass/Epoxy composites. As can be seen in the overlaps, the temperature increases initially at relatively high rates, approximating 0.33 °C/s and 0.61 °C/s for 50 mm/min and 150 mm/min, respectively. When the feed switches from 50 to 150 mm/min, the initial rate increases by roughly two-fold, while the peak temperature for the highest feed still remains 19% below the peak temperature recorded for the lowest feed. This proves that heat generation sensitively depends on both cutting conditions and material structure, which appears to interact closely during milling. After the initial rise, the temperature increment becomes non-linear before the ascent to the maximum value, after which the temperature starts to fall, indicating the cooling step.

The temperature recorded during the milling of a Glass/(Epoxy+SiO_2_) composite remained, overall, far below the glass transition temperature of epoxy, i.e., 77 °C, since it did not exceed 49 °C. This result promotes the use of SiO_2_ particles to effectively attenuate the heat localization and thermal damage that can occur during severe cutting conditions.

#### 3.3.2. Glass/(Polyester+SiO_2_)

**Figure 7:**
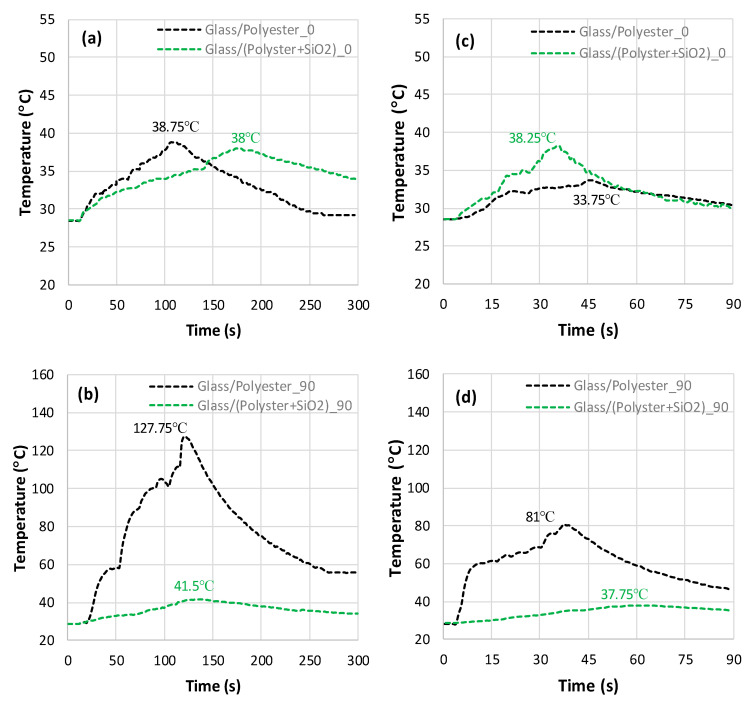
Temperature plots obtained from milling SiO_2_-free and SiO_2_-filled Glass/polyester composites. (**a**) Glass/Polyester, f=50 mm/min; θ=0°; (0.25 °C ≤ SD ≤ 1.79 °C). (**b**) Glass/Polyester, f=50 mm/min; θ=90°, (1.05 °C ≤ SD ≤ 2.89 °C). (**c**) Glass/Polyester, f=150 mm/min; θ=0°; (1.17 °C ≤ SD ≤ 2.2 °C). (**d**) Glass/Polyester, f=150 mm/min, θ=90°; (1.45 °C ≤ SD ≤ 2.59 °C).

The temperature histories recorded during milling of a Glass/Polyester silica-filled composite parallel and perpendicular to the fiber orientations are intentionally plotted with those of a Glass/Polyester composite to highlight the effect of the SiO_2_ addition (Figure 7). As can be seen from the overlaps, the peak temperatures reached under the different conditions are very similar, regardless of the fiber orientation and feed rate. It should be noted that the peak values are in the range of 39.625 ± 1.875 °C. The variation in temperature for the composite is thus negligible.

However, when milling parallel to the fiber at 50 mm/min, the addition of SiO_2_ has an insignificant effect. Furthermore, it seems to favor a temperature increase at higher feed levels, namely, 150 mm/min. In this milling configuration (Figure 7a,c), the material removal process is impacted by SiO_2_, which encrusts the interface, thus sliding over the lateral surface of the fibers. When the feed rate increases from 50 mm/min to 150 mm/min, the temperature varies slightly, which suggests that the feed rate is insignificant when milling parallel to the fibers.

In contrast, when milling perpendicular to the fiber, the temperature decreases drastically with the addition of SiO_2_. Typically, the peak values drop by 67% and 53% when the feed switches from 50 mm/min to 150 mm/min, respectively (Figure 7b,d). Overall, incorporating SiO_2_ into the Glass/Polyester composite appears to be very effective at limiting the temperature increase, particularly when cutting perpendicular to the fiber orientation. This indicates that silicon dioxide plays a heat-balancing role in the cutting of Glass/Polyester. The maximum temperatures obtained during the milling of a Glass/Polyester composite filled with 28% SiO_2_ remained below 41.5 °C irrespective of the cutting direction and feed rate, which is 2.096 times lower than the glass transition point of the matrix.

### 3.4. Analysis of Heat Energy

**Figure 8:**
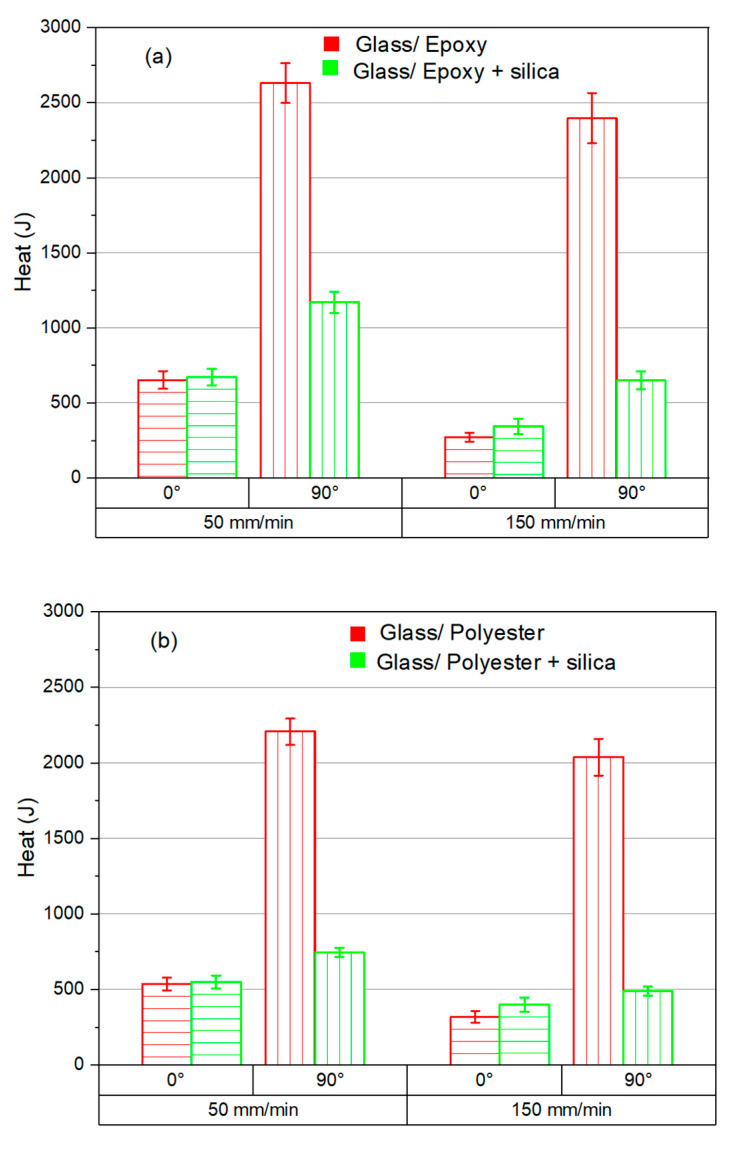
Heat stored from milling SiO_2_-free and SiO_2_-filled composites. (**a**) Glass/Epoxy and (**b**) Glass/Polyester.

In order to evaluate the effects of the addition of SiO_2_ on the thermal balance, cutting-induced heat was calculated based on Equation (8). Figure 8 illustrates the thermal energy generated during the milling of composite materials. The heat of both the SiO_2_-filled and SiO_2_-free composites is studied to assess the potential enhancement of the material behavior.

As shown in Figure 8a,b, the reliability of SiO_2_ in controlling milling-induced heat is evident regardless of the matrix type. However, the influence of silicon dioxide seems to be mitigated by the material removal process in the direction of the fiber. However, the reliability of SiO_2_ particles in drastically changing the heat energy balance appears obvious when cutting perpendicular to the fiber orientation. Figure 8a,b show a significant drop in heat energy obtained when milling perpendicular to the fiber direction. Among all the process parameters considered, i.e., feed rate, matrix type, and fiber orientation, the thermal behavior exhibits particular sensitivity to fiber orientation. Based on Figure 8a, it can be concluded that when milling perpendicular to the fiber direction, the addition of 28% SiO_2_ yields a drop in heat energy of 55% at a feed rate of 50 mm/min and of 15% at a feed rate of 150 mm/min. This decrease can be attributed to the properties of silicon dioxide.

On the one hand, SiO_2_ is able to dissipate energy via a heat conduction process since it possesses much higher thermal conductivity than glass fiber ($1.3–1.5\;W\cdot m^{-1}\cdot K^{-1}$ for SiO_2_ vs. $0.019–0.026\;W\cdot m^{-1}\cdot K^{-1}$ for glass fiber) and the matrices considered ($1.3–1.5\;W\cdot m^{-1}\cdot K^{-1}$ for SiO_2_ vs $.\;0.18–0.22\;W\cdot m^{-1}\cdot K^{-1}$ for matrices). On the other hand, it is deficient in terms of absorbing heat because of its relatively low heat capacity compared to glass fiber ($680–730\;J\cdot {kg}^{-1}\cdot K^{-1}$ for SiO_2_ vs. $835–840\;J\cdot {kg}^{-1}\cdot K^{-1}$ for glass fiber) and the matrices considered $(680–730\;J\cdot {kg}^{-1}\cdot K^{-1}$ for SiO_2_ vs. $1300–1930\;J\cdot {kg}^{-1}\cdot K^{-1}$ for matrices).

As for the milling of Glass/Polyester with and without the addition of SiO_2_ fillers, Figure 8b reflects a behavior similar to that observed for the Glass/Epoxy, while the addition of SiO_2_ leads to decreases in heat energy of approximately 15% and 36% for 50 and 150 mm/min, respectively.

### 3.5. Analysis of Surface Integrity

In order to evaluate how feed rate, together with material configuration, affects the quality of surface integrity, SEM observations were carried out on both the rough and the freshly machined surfaces of the composite samples studied.

#### 3.5.1. SiO_2_-Free Specimens

- As-Molded Matrix

**Figure 9:**
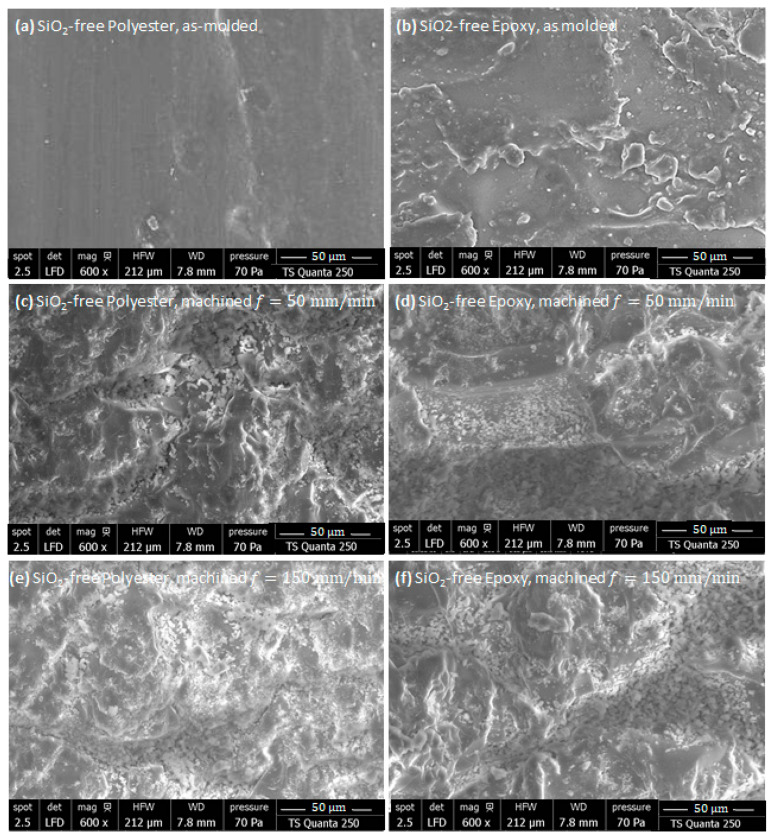
SEM micrographs of as-molded SiO_2_-free matrices (**a**) Polyester and (**b**) Epoxy; and SEM micrographs of fresh-milled SiO_2_-free matrices (**c**) Polyester, f=50 mm/min, (**d**) Epoxy, f=50 mm/min, (**e**) Polyester, f=150 mm/min, and (**f**) Epoxy, f=150 mm/min.

To establish a baseline for comparison, SEM inspections were first performed on non-machined samples. Figure 9 illustrates the initial surface features of the specimens in their as-molded state before the influence of edge-milling.

The surface morphology of the untrimmed pure-polyester sample (Figure 9a) appears remarkably smooth and homogeneous, with very limited irregularities. Only minor pores or shallow voids, attributable to the molding process, can occasionally be observed. The absence of pronounced topographic variations suggests that polyester, in its pure matrix form, exhibits a relatively stable solidification behavior with reduced shrinkage during polymerization.

In contrast to polyester, the pure-epoxy surface (Figure 9b) exhibits a slightly uneven morphology, characterized by localized three-dimensional features and irregular reliefs. These patterns are most likely linked to the molding conditions, particularly differential shrinkage during curing and possible mold surface imperfections. Such heterogeneities highlight the sensitivity of epoxy resins to thermal gradients and curing kinetics. Establishing this baseline morphology is crucial for distinguishing between inherent molding-induced irregularities and additional damage generated by milling operations.

- As-Machined Matrix

The SEM images of the machined surfaces displayed in Figure 9c–f reveal more irregular topographies compared to the as-molded state, reflecting the effects of machining with an abrasive tool. The nature of the abrasive tool significantly influences the surface integrity. Its interaction with the matrix generates micro-asperities, striations, and localized material removal, whose severity depends on both the matrix type and the feed rate.

In fact, epoxy specimens exhibit superior surface integrity, with fewer micro-cracks and less pronounced micro-chipping along the machined edges. In contrast, polyester matrices show more pronounced roughness, larger and deeper micro-chippings, and longer cracks, indicating a higher susceptibility to brittle fracture under abrasive cutting. The influence of feed rate is also evident: surfaces machined at 150 mm/min display more severe irregularities and longer cracks, while those cut at 50 mm/min are comparatively smoother. Importantly, no evidence of thermal damage, such as burns or resin degradation, was observed in either material, suggesting that the milling parameters employed with the abrasive tool did not induce significant heat-affected zones.

- SiO_2_-Free Composites As-Machined Parallel to Fiber

As outlined above, energy demand and dissipative phenomena are strongly dependent on fiber orientation during the milling of composites. To investigate the combined effects of fiber orientation and feed rate on surface integrity and process-induced damage, SEM inspections were conducted on freshly trimmed edges of Glass/Epoxy and Glass/Polyester composites parallel to fiber orientation.

**Figure 10:**
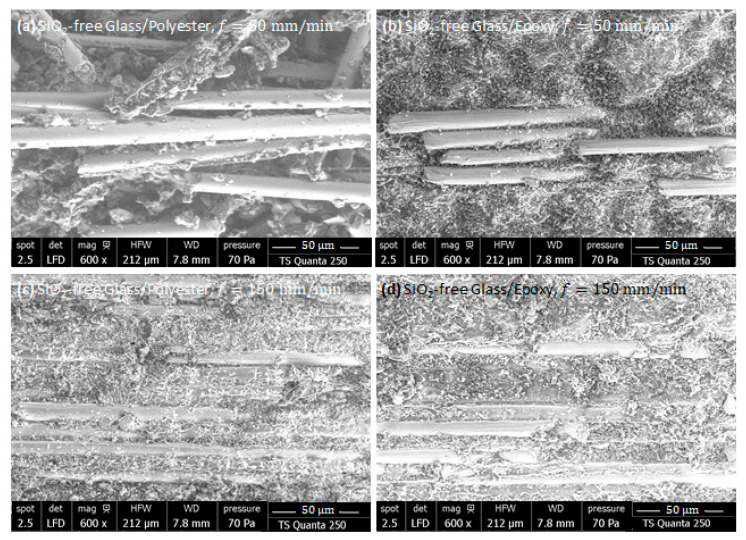
SEM micrographs of fresh surface of SiO_2_-free composites, obtained by milling parallel to fiber: (**a**) Glass/Polyester, f=50 mm/min, (**b**) Glass/Epox, f=50 mm/min (**c**) Glass/Polyester, f=150 mm/min, and (**d**) Glass/Epoxy, f=150 mm/min.

Under a feed rate of 50 mm/min (Figure 10a,b), SEM inspections of the machined edges reveal insufficient bonding between the glass fibers and the resin, with a predominance of interfacial failures such as long, uncovered fibers, separated fragments, dusty debris, and clear evidence of polymer detachment. These phenomena are more pronounced in polyester-based composites due to their lower interfacial toughness. The observed damage is generated by the combined actions of cyclic friction and fiber pull-out, since the abrasive tool remains in contact with the fiber surface over a relatively long period. This extended interaction promotes higher mechanical and thermal energy dissipation at the tool–composite interface. Owing to the poor thermal conductivity of glass fibers, heat localizes at the fiber–matrix boundary, accelerating interfacial degradation and favoring premature debonding. Consequently, surface integrity is severely compromised, as reflected by the extensive fiber exposure, reinforcement fragmentation, and matrix loss, with the extent of the damage being consistently higher in Glass/Polyester than in Glass/Epoxy.

When machining at 150 mm/min (Figure 10c,d), SEM examinations reveal a remarkable reduction in interfacial degradation. The glass fibers remain more effectively embedded within the resin, and the generated chips predominantly appear as fiber–matrix agglomerates rather than isolated debris or fine dust. This behavior can be attributed to the shorter tool–workpiece interaction time, which limits heat accumulation at the fiber–matrix boundary. As a result, the dominant removal mechanisms switch to abrasive cutting and frictional shearing, thereby favoring mechanical energy dissipation to the detriment of thermal energy dissipation. Consequently, surface integrity is relatively better preserved under high-feed conditions, with reduced interfacial losses and less pronounced fiber exposure than those observed at lower feed rates.

- SiO_2_-Free Composites As-Machined Perpendicular to Fiber

**Figure 11:**
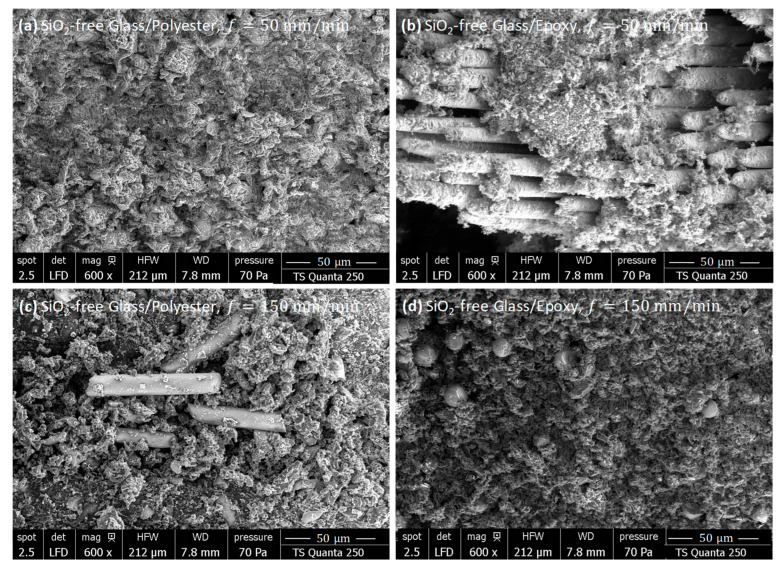
SEM micrographs of fresh surface of SiO2-free composites, obtained by milling composites perpendicular to fiber direction: (**a**) Glass/Polyester, f=50 mm/min, (**b**) Glass/Epoxy, f=50 mm/min, (**c**) Glass/Polyester, f=150 mm/min, and (**d**) Glass/Epoxy, f=150 mm/min.

At a 50 mm/min feed rate, SEM inspections of cut surfaces when milling perpendicular to the fiber highlight a pronounced difference in surface integrity between the two composite types (Figure 11a,b). For Glass/Epoxy, the machined edges exhibit pronounced interfacial failures with deep matrix loss surrounding fiber cross-sections, producing fractured fibers and dusty, fragmented chips. These damages result from both shearing and heat accumulation at fiber–matrix interfaces, as glass fibers impede thermal diffusion and act as barriers to heat transfer. Consequently, temperature increases locally, accelerating matrix degradation and fiber debonding. In Glass/Polyester composites, more severe mechanisms occur due to the lower interfacial toughness of polyester, leading to fragmented debris. The relatively high cutting time extends tool–material interaction, which favors cyclic friction and pull-out, dominates the material removal process, and critically compromises surface integrity.

At a 150 mm/min feed rate (Figure 11c,d), SEM observations reveal significantly improved surface integrity for both G/E and G/P composites. Fibers remain partially embedded, and chips predominantly consist of cohesive fiber–matrix fragments rather than isolated debris or dust. The reduced contact time between the abrasive tool and the composite surface limits localized heating, thereby mitigating thermally induced interfacial degradation. As a result, the material removal process is primarily governed by mechanical energy dissipation through abrasive cutting and shearing. Surface damage is comparatively less severe, with fewer uncovered fibers and more uniform matrix coverage around the reinforcement. The distinction between Glass/Epoxy and Glass/Polyester is maintained, but both benefit from the shift toward mechanically dominated removal mechanisms, highlighting the critical role of feed rate in controlling thermomechanical effects and preserving surface integrity.

#### 3.5.2. SiO_2_-Filled Composites

**Figure 12:**
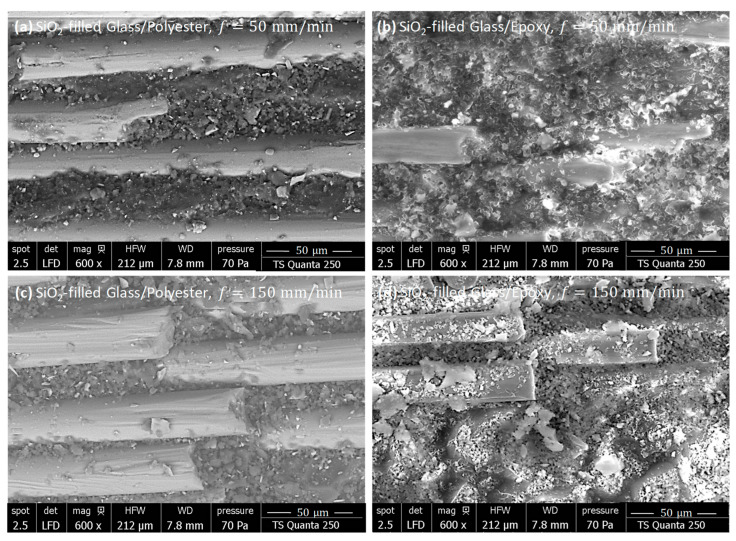
SEM micrographs showing surface features of SiO_2_-filled GFRP samples milled parallel to the fiber orientation. (**a**) Glass/(Polyester+SiO_2_), f=50 mm/min, (**b**) Glass/(Epoxy+SiO_2_), f=50 mm/min, (**c**) Glass/(Polyester+SiO_2_), f=150 mm/min, and (**d**) Glass/(Epoxy+SiO_2_), f=150 mm/min.

**Figure 13:**
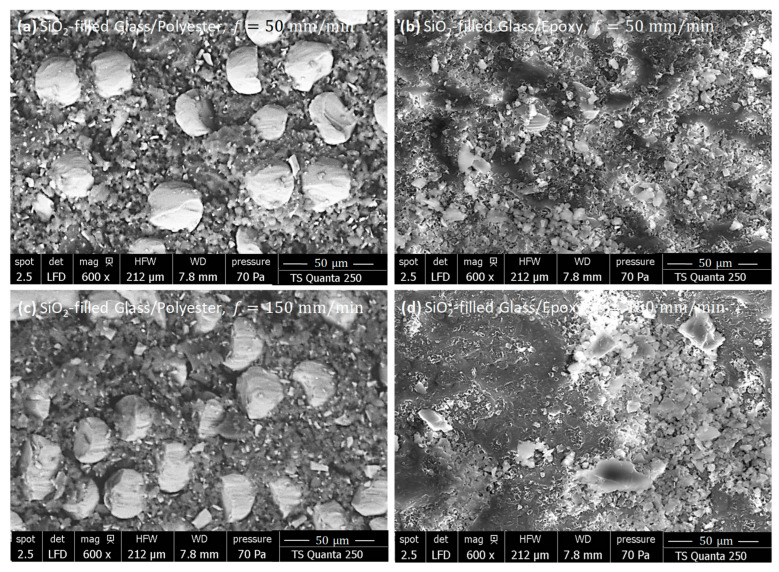
SEM observations showing the surface morphology of SiO_2_-filled GFRP composites obtained by milling perpendicular to the fiber orientation (**a**) Glass/(Polyester+SiO_2_), f=50 mm/min, (**b**) Glass/(Epoxy+SiO_2_), f=50 mm/min, (**c**) Glass/(Polyester+SiO_2_), f=150 mm/min, and (**d**) Glass/(Epoxy+SiO_2_), f=150 mm/min.

To further evaluate the effects of silicon dioxide on surface integrity, SEM inspections were performed on freshly machined SiO_2_-filled composites with specified fiber orientations. Figure 12 and Figure 13 present SEM micrographs of cut surfaces obtained by milling parallel and perpendicular to the fiber orientation, respectively. The investigations include specimens machined at both 50 and 150 mm/min.

- SiO_2_-Filled Composites As-Milled Parallel to Fiber

At a low feed rate (Figure 12a,b), SEM micrographs revealed a significant improvement in the integrity of the machined surfaces compared to non-filled composites, regardless of the matrix type. Fibers remained mostly aligned and embedded within the matrix, with limited fragmentation, which reflects a stable fiber–matrix adhesion. This is clearer in SiO_2_-filled Glass/Polyester specimens. Indeed, the presence of SiO_2_ promoted a consolidated microstructure, limiting extensive fiber exposure and enhancing resistance to thermal and mechanical degradation. Dispersed SiO_2_ grains (~12 μm in polyester and ~5 μm in epoxy) contributed to surface refinement and debris approximating the same average size.

The SEM micrographs also showed that the fillers influenced chip morphology and energy dissipation. The chips appeared flattened and heterogeneous, with cavities corresponding to extracted silica grains, which interacted with both the resin and the fibers, promoting localized micro-grinding and the controlled fracture of glass reinforcements. This interaction, together with the mass, hardness, and volume fraction of the fillers, redistributed the heat within the cutting zone, limiting interfacial overheating and stabilizing tribological conditions. While fiber orientation remained important, the polyester-based matrix better preserved fibers than the epoxy-based matrix, indicating higher interfacial resilience under combined abrasive and filler-induced stresses. Overall, these observations demonstrate that SiO_2_ fillers efficiently preserve surface integrity, optimize stress transfer, and enhance thermal stability during the machining of composites parallel to the fiber.

At a 150 mm/min feed rate (Figure 12c,d), SEM inspections showed a more complex surface morphology. In polyester-based composites, fractured fibers were observed alongside matrix fragments, while both polyester and epoxy samples exhibited dispersed debris, reflecting local micro-damage attributed to localized loading around the SiO_2_ particles. SiO_2_ grains looked slightly larger than at the low feed rate, with approximate sizes of 17 μm in polyester and 9 μm in epoxy, and they were often found in mixtures with ground matrix and fiber fragments, resulting in a combined micro-grinding effect. Hiatuses and small cavities were particularly evident in polyester, owing to the localized interactions of the constituents. Despite these alterations, no burning signature or thermal degradation was detected, confirming that the fillers effectively prevent excessive heat generation. Compared with non-filled specimens, the surface remained rougher due to the presence of the debris and dust of various constituents, while overall integrity was still better preserved, highlighting the reinforcing role of SiO_2_. These observations also indicate that a relatively low feed rate is more favorable for cutting SiO_2_-filled GFRP parallel to fiber, as it results in less mechanical degradation while preserving the thermal benefits of silicon dioxide.

- SiO_2_-Filled Composites As-Milled Perpendicular to Fiber

When machining perpendicular to fiber orientation, the behavior of fresh surfaces seems to depend, with some sensitivity, on the matrix nature. Overall, SiO_2_-filled polyester specimens displayed a more homogeneous and consolidated surface morphology than SiO_2_-filled epoxy specimens. The SiO_2_-filled Glass/Epoxy composites (Figure 13a,b) showed a significantly deteriorated surface.

The perpendicular fiber orientation promoted severe interaction with the abrasive tool, leading to severe crushing and near-complete leveling of the fibers at the surface. SEM micrographs revealed that the cut area consisted mainly of mixed debris formed by finely ground silica particles, matrix fragments, and pulverized fibers, with almost no intact reinforcement visible. This effect was accentuated at the higher feed rate (150 mm/min), where the debris appeared coarser and more agglomerated than at 50 mm/min, entailing enhanced fragmentation and heat localization.

In contrast, the fibers kept a regular diameter and remained well-embedded within the surrounding matrix during the milling of SiO_2_-filled Glass/Polyester (Figure 13c,d). The fiber–matrix interface appeared compact and continuous, indicating stable mechanical and thermal behavior. Only local streaks of micro-debonding were observed, confirming that the SiO_2_ fillers efficiently stabilized the fiber–matrix junction and limited excessive surface degradation. However, increasing the feed rate slightly affected the fiber appearance and interface quality, resulting in better resilience to mechanical stresses and improved resistance to thermal degradation. These observations confirm that SiO_2_ fillers, combined with the inherent toughness of the polyester matrix, promote superior surface integrity and a more stable machining response in SiO_2_-filled Glass/Polyester compared to SiO_2_-filled Glass/Epoxy laminates.

## 4. Conclusions

The investigation of the thermal behavior of glass fiber-reinforced SiO_2_-filled polymers when dry milling is specially addressed in this study. The key conclusions derived from the experimental findings are drawn below.

- Fiber orientation significantly affects the temperature and heat generated during the milling. Severe temperatures, exceeding the glass transition point of the matrix, are typically recorded in SiO_2_-free specimens when milling perpendicular to the fiber orientation.

- SiO_2_-filled matrices effectively reduce peak temperatures (by about 67%) and limit heat accumulation, supporting improved surface integrity under dry milling, particularly under the most severe cutting conditions, i.e., θ=90° − f=50 mm/min. The SiO_2_ particles promote better heat management that keeps the temperatures sufficiently low relative to the glass transition point.
- Increasing the feed rate—that is, from 50 to 150 mm/min—causes the temperature to drop. The higher feed rate limited the heat buildup due to the short contact time between the tool and the specimen.
- Energy-based heat metrics complement peak temperature and provide a more physically representative measure of thermal severity.
- Under similar conditions, cutting Glass/Polyester composites yields lower heat compared to Glass/Epoxy composites since polyester possesses specific heat at a significantly higher rate than epoxy.
- Ultimately, the reduction in peak temperatures below the glass transition point of the matrix by the addition of SiO_2_ fillers is an important practical outcome for emergent applications where temperature has sensitive effects on the behavior of FRP structures.

## Figures and Tables

**Table 1:** Properties of resinous matrices used for specimen preparation.

Properties	Epoxy 1050	Polyester TP280
Appearance	Opalescent	Clear blue
$\mathrm{Density}\;\rho \;(g\cdot {cm}^{-3})$	1.27 ± 0.05 [27]	1.10 ± 0.05 [27]
$\mathrm{Specific}\;\mathrm{heat}\;c_{p}(J/(Kg\cdot K)$)	1300 [28,29]	1870–1930 [30,31]
Thermal conductivity λ(W/m⋅K)	0.2–0.22 [29,32,33]	0.181 ± 0.003 [34,35]
$\mathrm{Glass}\;\mathrm{transition}\;\mathrm{point}\;T_{g}(℃)$	77 [27]	87 [27]
Viscosity κ (Pa⋅s)	1.043 [27]	0.647 [27]
Dry extract (% volume)	33% [27]	31% [27]
Gel time, at 23 °C (min)	210 (23 °C with 30% of 1055S)	30 (25 °C with 2% of MEKP)

**Table 2:** Properties of SiO_2_ silica grains and E-glass fiber used in specimen preparation.

Properties	SiO_2_	Glass Fiber
Density $\rho \;(g\cdot {cm}^{-3})$	1.73–2.65 [36,37]	2.48 [38]
Specific heat $c_{p}(J\cdot {kg}^{-1}\cdot K^{-1}$)	680–730	835–840 [39,40]
Thermal conductivity $\lambda (W\cdot m^{-1}\cdot K^{-1})$	1.3–1.5	0.019–0.026 [32,39]
Thermal expansion $\alpha \;(10^{-6}\cdot K^{-1})$	0.55–0.75	4.73–6.52 [38]
Average sub-angular size (μm)	300 [37]	
Average diameter (μm)		19.27–24.70 [27]

**Table 3:** Thermal conductivities of considered composites in $\left(W\cdot m^{-1}\cdot K^{-1}\right)$.

Composite	Volume Fraction	$\lambda_{∥}$	$\lambda_{⊥}$
Glass/Epoxy	$V_{f}=0.15$ $V_{m}=0.85$	0.182 ± 0.009	0.093 ± 0.007
Glass/(Epoxy+SiO_2_)	$V_{f}=0.15$ $V_{m}=0.57$ $V_{s}=0.28$	0.515 ± 0.066	0.104 ± 0.013
Glass/Polyester	$V_{f}=0.15$ $V_{m}=0.85$	0.156 ± 0.003	0.088 ± 0.004
Glass/(Polyester+SiO_2_)	$V_{f}=0.15$ $V_{m}=0.57$ $V_{s}=0.28$	0.498 ± 0.030	0.100 ± 0.010

**Table 4:** Statistical parameters obtained from the polynomial fitting of temperature data.

Feed (mm/min)	Material	Cutting Test	SD	RMSE	MAE	R^2^
50	Epoxy	Pure Epoxy	0.5514	0.5496	0.3595	0.9827
		Glass/Epoxy_0°	0.2162	0.2154	0.1426	0.9945
		Glass/Epoxy_90°	1.6416	1.6365	1.0430	0.9958
		Glass/(Epoxy+SiO_2_)_0°	0.3775	0.3762	0.2758	0.9908
		Glass/(Epoxy+SiO_2_)_90°	0.2739	0.2730	0.2143	0.9976
	Polyester	Pure Polyester	0.4459	0.4426	0.3439	0.9852
		Glass/Polyester_0°	0.2039	0.2033	0.1551	0.9955
		Glass/Polyester_90°	2.7865	2.7777	2.0978	0.9882
		Glass/(Polyester+SiO_2_)_0°	0.1159	0.1154	0.0899	0.9978
		Glass/(Polyester+SiO_2_)_90°	0.1832	0.1827	0.1361	0.9973
150	Epoxy	Pure Epoxy	0.4583	0.4265	0.3947	0.9949
		Glass/Epoxy_0°	0.1645	0.1639	0.1191	0.9909
		Glass/Epoxy_90°	1.6284	1.6226	1.0048	0.9948
		Glass/(Epoxy+SiO_2_)_0°	0.0906	0.0902	0.0695	0.9974
		Glass/(Epoxy+SiO_2_)_90°	0.1909	0.1902	0.1536	0.9956
	Polyester	Pure Polyester	0.8580	0.8553	0.5538	0.9789
		Glass/Polyester_0°	0.1154	0.1151	0.0758	0.9938
		Glass/Polyester_90°	1.5199	1.5152	1.0142	0.9888
		Glass/(Polyester+SiO_2_)_0°	0.2881	0.2867	0.2231	0.9877
		Glass/(Polyester+SiO_2_)_90°	0.1292	0.1285	0.0992	0.9983

## Data Availability

The original contributions presented in the study are included in the article. Further inquiries can be directed to the corresponding author.
